# Synovial calprotectin for the diagnosis of periprosthetic joint infection: a diagnostic meta-analysis

**DOI:** 10.1186/s13018-021-02746-2

**Published:** 2022-01-04

**Authors:** Xinyu Peng, Haitao Zhang, Pengfei Xin, Guowen Bai, Yingjie Ge, Miaoxin Cai, Rui Wang, Yueguang Fan, Zhihui Pang

**Affiliations:** 1 The First Clinical Medical School, Guangzhou University of Chinese Medicine, Jichang Road 12#, District Baiyun, Guangzhou, Guangdong China; 2 Department of Orthopaedics, The First Affiliated Hospital of Guangzhou University of Chinese Medicine, Jichang Road 16#, District Baiyun, Guangzhou, 510405 Guangdong China

**Keywords:** Periprosthetic joint infection, Calprotectin, Diagnosis, Meta-analysis

## Abstract

**Background:**

Periprosthetic joint infections (PJI) are a rare but severe complication of total joint arthroplasty (TJA). However, the diagnosis of PJI remains difficult. It is one of the research that focuses about diagnosis for PJI for majority researchers to discover a novel biomarker. This meta-analysis tried to evaluate diagnostic value of synovial calprotectin for PJI.

**Methods:**

This meta-analysis search of the literature was conducted in PubMed, EMBASE, Web of Science, and the Cochrane Library. Literature quality was appraised using Quality Assessment of Diagnostic Accuracy Studies (QUADAS-2) based on RevMan (version 5.3). The diagnostic value of calprotectin for PJI was evaluated by calculating sensitivity, specificity, positive likelihood ratio (PLR), negative likelihood ratio (NLR), diagnostic odds ratio (DOR), diagnostic score and area under SROC (AUC) based on the Stata version 14.0 software. We conduct subgroup analysis according to the study design, cutoff values, the country of study, and gold standard.

**Results:**

Seven studies were included in this meta-analysis. The pooled sensitivity of synovial calprotectin for the diagnosis of PJI was 0.94 (95% CI, 0.87–0.98), and the specificity was 0.93 (95% CI, 0.87–0.96). The pooled AUC, PLR, and NLR for synovial calprotectin were 0.98 (95% CI, 0.96–0.99), 13.65 (95% CI, 6.89–27.07), and 0.06 (95% CI, 0.02–0.15), respectively. The pooled diagnostic score and DOR were 5.4 (95% CI, 3.96–6.85) and 222.32 (95% CI, 52.52–941.12), respectively.

**Conclusion:**

In summary, this meta-analysis indicates that synovial calprotectin is a promising biomarker of assistant diagnosis for PJI, as well as recommended test for excluding diagnostic tool.

**Supplementary Information:**

The online version contains supplementary material available at 10.1186/s13018-021-02746-2.

## Introduction

With the development of social economy, the caseload of primary and revision hip and knee arthroplasty surgeons is gradually increasing [1]. Periprosthetic joint infections (PJI) are a rare but severe complication of total joint arthroplasty (TJA) [2], with a prevalence ranging between 0.7 and 2.4% [3]. PJI and concomitant revision arthroplasty exert enormous pressure on surgeons, healthcare institutions, and especially patients [4–6]. Accurate diagnosis of PJI is very difficult for surgeons on early stage, because the symptoms are atypical and identified difficultly. For a long time, a lack of standardized diagnostic criteria is the barrier to manage PJI for surgeons and researchers [7]. Several groups, such as the American Academy of Orthopedic Surgeons (AAOS), Musculoskeletal Infection Society (MSIS), the European Bone and Joint Infection Society (EBJIS) and Infectious Diseases Society of America (IDSA), have been committed to make the diagnostic criteria of PJI perfect [8, 9]. Although the newest criteria after International Consensus Meeting (ICM) in 2018 have been published for PJI, the diagnosis of PJI is still challenging [10]. The 39th Annual Meeting of EBJIS will be held this year. The diagnosis of PJI is reliant upon laboratory examinations, and therefore a growing number of researchers lay emphasis on finding more valuable and effective biomarkers for detection of PJI [11, 12].

Calprotectin, a 36 kDa zinc and calcium binding protein, is mostly released by neutrophilic granulocytes and monocytes/macrophages, when receiving inflammatory stimulation [13, 14]. It is shown that calprotectin can promote inflammation reaction and is regarded as the detection of inflammatory markers for rheumatoid arthritis (RA), inflammatory bowel disease (IBD), and spondyloarthritis [15–18]. Recent follow-up studies suggest that the synovial calprotectin was a useful biomarker for the diagnosis of PJI [19].

The aim of this study was to systematically review the literature regarding calprotectin concentrations in synovial fluid of joint with PJI and to investigate diagnostic value of calprotectin in PJI.

## Materials and methods

Our research was conducted in accordance with the Preferred Reporting Items for Systematic Reviews and Meta-Analyses (PRISMA) guidelines [20].

### Search strategy

This meta-analysis systematically searched all literature about calprotectin in the diagnosis of PJI in PubMed, EMBASE, Web of Science, and Cochrane Library (all up to June 2021), only including English literature. Combinations of the terms, “periprosthetic joint infection” or “prosthesis-related infections” and “calprotectin,” were used. Moreover, we searched manually the references of the included research and related literature on calprotectin to gain the literature that we needed for this study (Additional file 1).

### Inclusion and exclusion criteria

Two researchers reviewed independently the title and abstract of each assay to select those that were likely for further screening. When two researchers were in the face of disagreement, they were going to submit the problem to Professor Zhihui Pang and the final result depends on him. Inclusion/exclusion criteria were established before the literature search. The following inclusion criteria were applied: (1) using calprotectin as an index for the diagnosis of PJI. (2) Sufficient data can be extracted for the construction of a 2 × 2 contingency table. Exclusion criteria were: insufficient data to calculate sensitivity and specificity; case reports, commentaries, expert opinion, and narrative reviews; duplicates.

### Data extraction and quality assessment

Two reviewers (Yingjie Ge, Miaoxin Cai) extracted information independently from each study, using a standardized data extraction form, which included general study characteristics and patients’ clinical characteristics [author, year of publication, country, the design type of the study, number of patients and controls, age and body mass index (BMI) of patients and controls, the gold standard used in the study, the detection method, and the cutoff value of calprotectin]. It was very vital for them to extract information false/true positive, false/true negative from 2 × 2 table for diagnostic studies, sensitivity, and specificity.

We appraised the quality of all the literature included studies according to the Quality Assessment of Diagnostic Accuracy Studies (QUADAS-2) in the RevMan (version5.3).

### Statistical analysis and heterogeneity assessment

Stata 14.0 software was used to conduct the statistical analysis of the study. The combination of the sensitivity, specificity, positive likelihood ratio (PLR), negative likelihood ratio (NLR), diagnostic odds ratio (DOR), summary receiver operating characteristic (SROC) curve, and the area under the curve (AUC) was based on the bivariate model by the “Midas” command. The diagnostic accuracy of calprotectin was assessed by AUC. Additionally, the heterogeneity was estimated by calculating the inconsistency index (*I*^2^) statistic.

The value of *I*^2^ was a range from 0 to 100%; *I*^2^ value of 75%, 50%, and 25% indicates high, moderate, and low inconsistency, respectively. When there was high heterogeneity, we identified the source of heterogeneity by subgroup analysis. If statistical heterogeneity was remarkable, we found the potential sources of bias from the variables, such as the type of study design, cutoff value, the number of cases, diagnostic standard, and the country or region. The publication bias was estimated by drawing the funnel chart (Deeks’ funnel plot). Moreover, the Fagan plot diagram was used to reveal the change of the diagnostic value of calprotectin.

## Results

### Search results and study characteristics

**Fig. 1:**
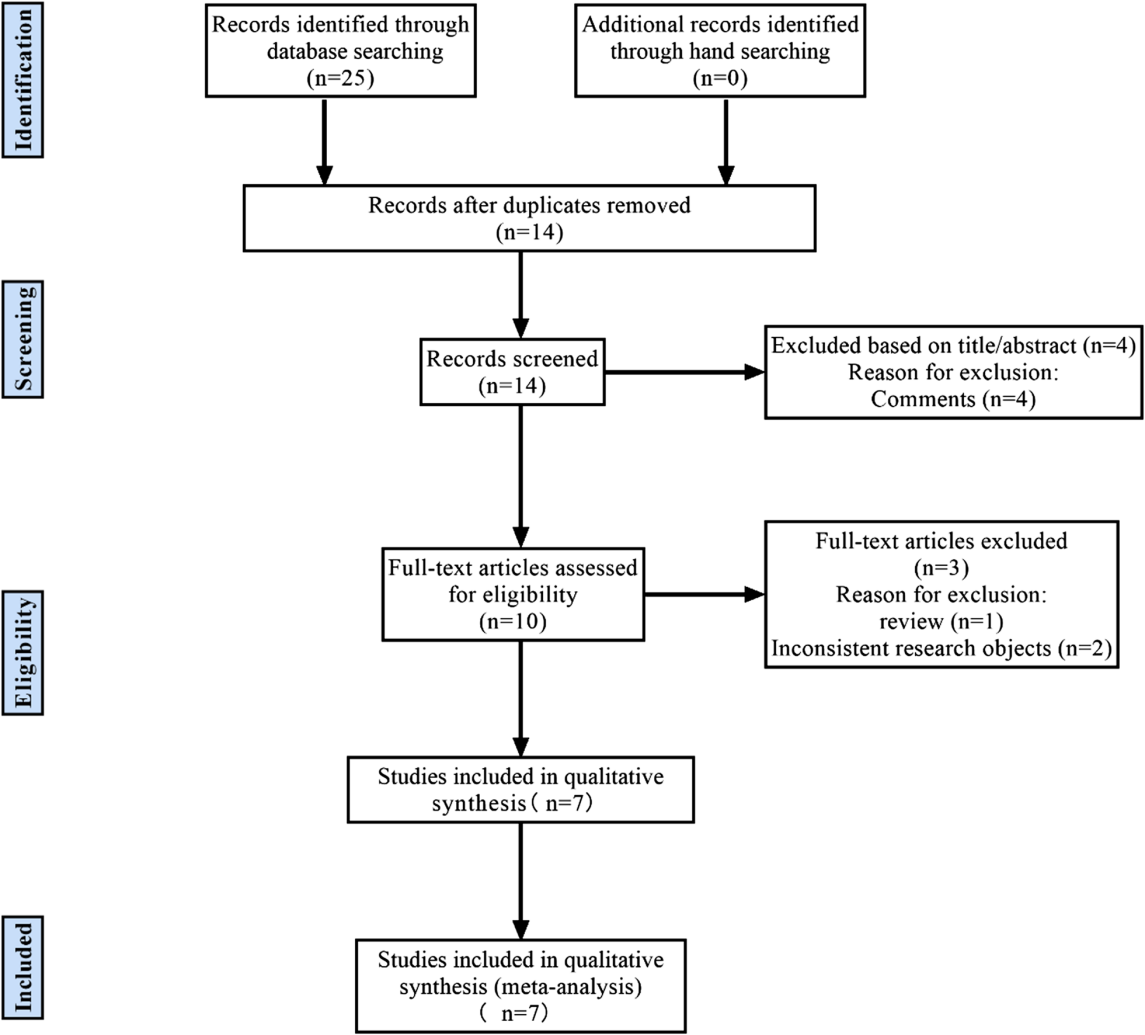
Study flowchart

The flowchart about the study selection process is shown in Fig. 1. The electronic database search yielded a total of 25 studies (11 in PubMed, 7 in EMBASE, 6 in Web of Science, and 1 in the Cochrane Library). After excluded 11 duplicates, 14 studies remained. Four studies were removed by scanning titles and abstracts. After reading the full text in detail, the meta-analysis included 7 studies finally [19, 21–26].

The 7 studies consisted of 525 patients who had undergone hip, knee, shoulder, or elbow joint replacement, including 226 patients with PJI and 299 patients with non-PJI. Two studies used tests were dedicated to synovial fluid, one dedicated to plasma samples and dedicated 4 to fecal samples. Six studies were conducted prospectively and one retrospectively. Four studies applied the Musculoskeletal Infection Society (MSIS) as the diagnosis standard, and 3 applied International Consensus on Infection (ICM). Detailed characteristics and the main results of each study are shown in Tables 1 and Table 2, respectively.

**Table.1:** Characteristics of the studies in meta-analysis for the diagnosis of PJI applying Calprotectin

Study	Year	Country	Study design	Gender (M/F)	Mean age*	BMI*	Detection method	Cutoff values	Gold standard
Dariusz Grzelecki et al	2021	Poland	P	25/60	68.3 / 65.5	29.3/ 29.9	Immunoturbidimetric Calprotectin Immunoassay (GCal, Gentian, Moss, Norway)	1.5 mg/L	ICM (2018)
Jared Warren et al	2021	USA	P	57/66	65.4 / 66.9	32.0/ 34.2	Lyfstone Calprotectin Point of Care Test Kit (Lyfstone)	50 mg/L	MSIS (2013)
Alexander J Trotter et al	2020	TUK	R	37/32	NA	NA	The Lyfstone calprotectin test (Lyfstone AS, Tromsø, Norway)	14 mg/L	ICM (2018)
Marjan Wouthuyzen-Bakker et al	2017	Netherlan-ds	P	25/36	60/65	NA	The Rapid Calprotectin High Range Quantum Blue Assay (BÜHLMANN laboratories AG)	50 mg/L	MSIS (2011)
Marjan Wouthuyzen-Bakker et al	2018	Netherlan-ds	P	NA	NA	NA	The Rapid Calprotectin High Range Quantum Blue Assay (BÜHLMANN laboratories AG)	50 mg/L	MSIS (2011)
Paolo Salari et al	2019	Italy	P	36/40	NA	NA	Calprest NG (Eurospital, Trieste, Italy)	50 mg/L	ICM (2018)
Zeyu Zhang et al	2019	China	P	20/43	NA	NA	ELISA (Hycult Biotech, Uden, the Netherlands)	173 ug/ml	MSIS (2013)

^*^The values were given as the number with Non-PJI / PJI, P prospective study, R retrospective study, NA not applicable

**Table.2:** Data extracted for the construction of 2 × 2 table

Author	Year	TP	FP	FN	TN	Total
Dariusz Grzelecki et al	2021	43	2	2	38	85
Jared Warren et al	2021	52	3	1	67	123
Alexander J Trotter et al	2020	18	11	6	34	69
Marjan Wouthuyzen-Bakker et al	2017	17	4	2	38	61
Marjan Wouthuyzen-Bakker et al	2018	13	3	2	34	52
Paolo Salari et al	2019	28	2	0	42	72
Zeyu Zhang et al	2019	20	1	1	41	63

TP True positive; FP False positive; FN False negative; TN True negative

### Quality and publication biases of the included studies

**Fig. 2:**
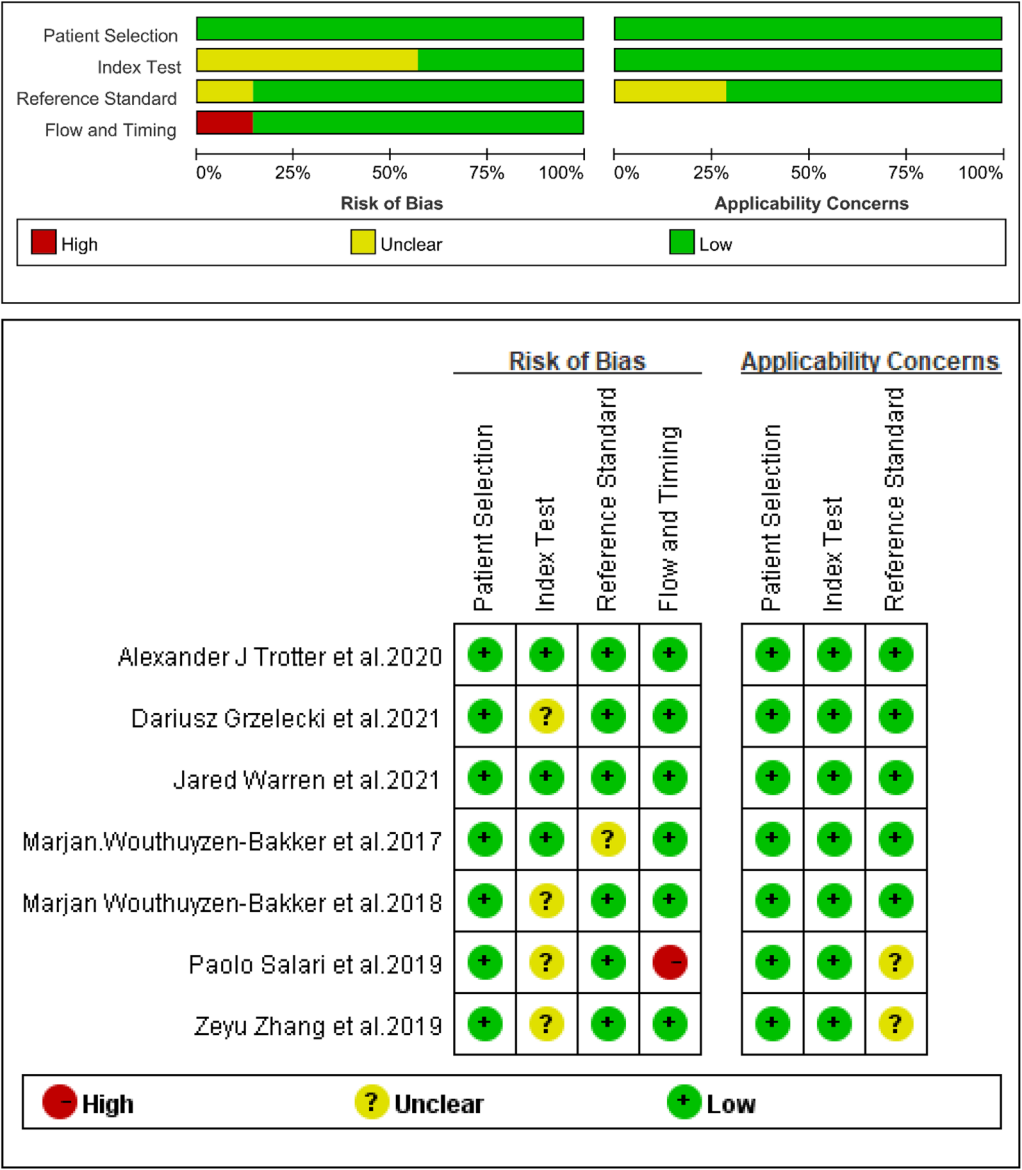
Quality assessment of included studies based on QUADAS-2 tool criteria

**Fig. 3:**
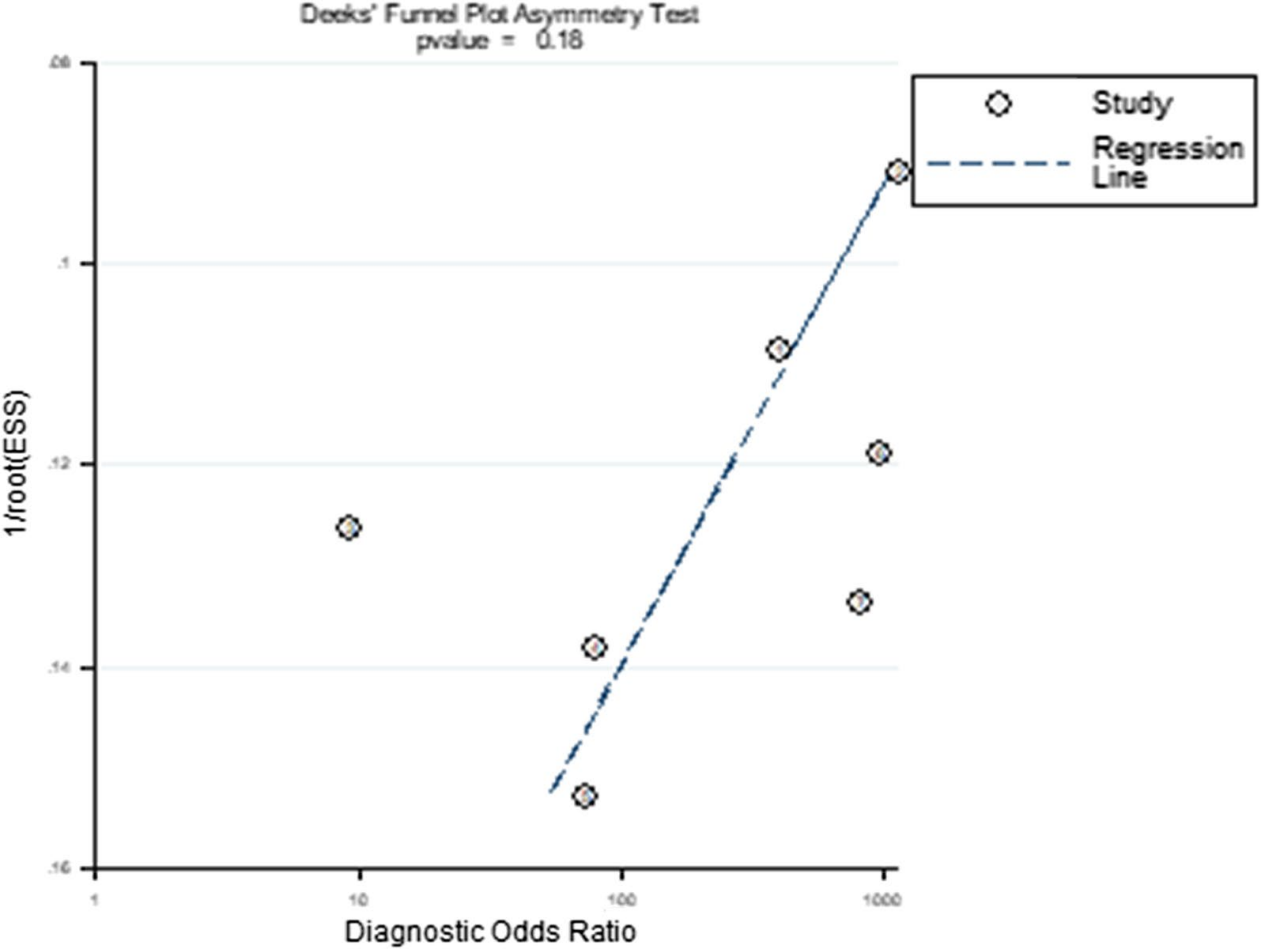
Funnel plot for publication bias assessment of included studies

Through the QUADAS-2 scale, the quality of 7 included studies was assessed (Fig. 2). The publication bias for calprotectin was detected by evaluating funnel plot asymmetry according to the Deeks tests. The result of publication bias is shown in Fig. 3 and indicated no significant publication bias for calprotectin (*P* = 0.18 > 0.1).

### Diagnostic value of calprotectin for PJI

**Fig. 4:**
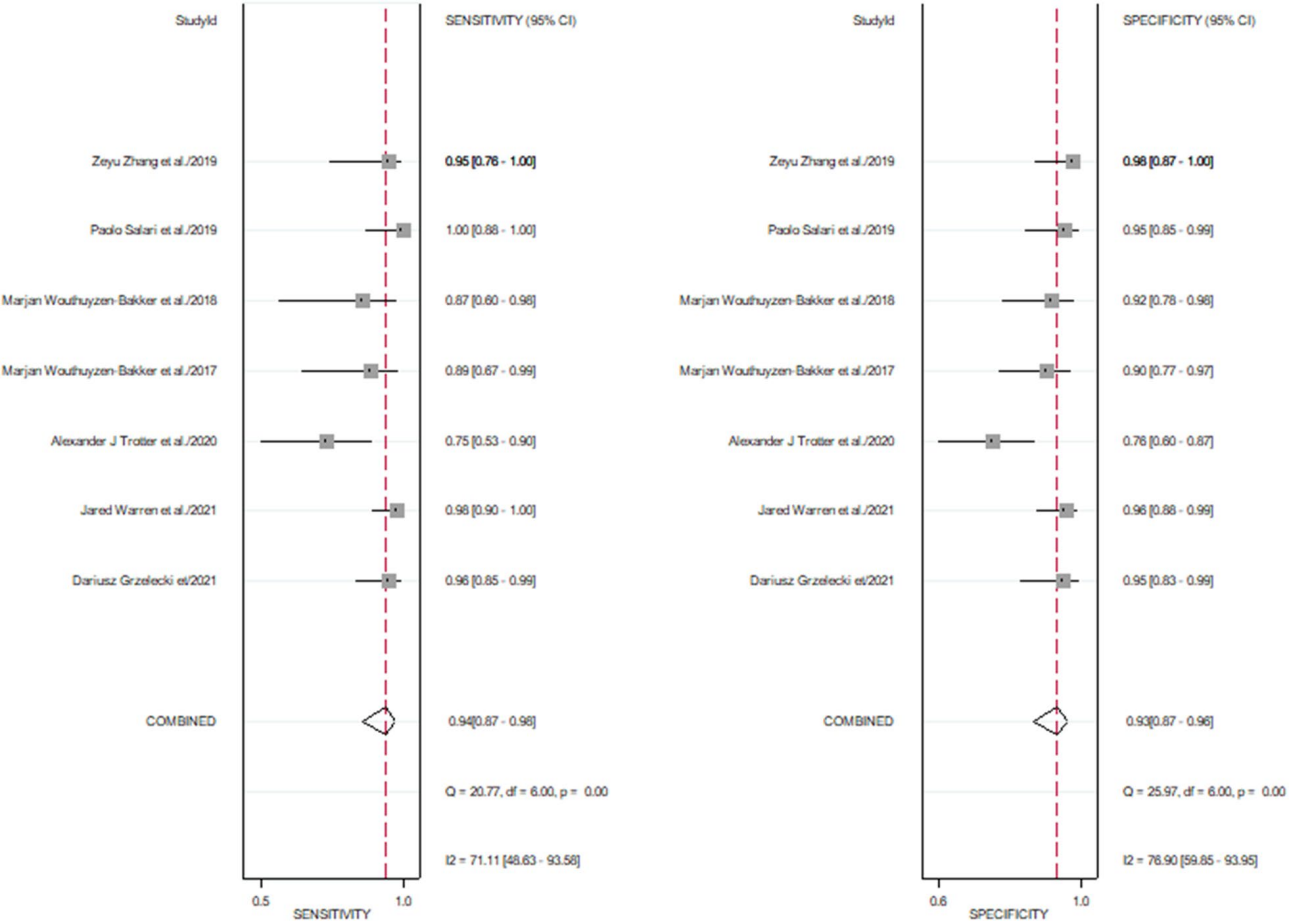
Pooled sensitivity and specificity of calprotectin for PJI

**Fig. 5:**
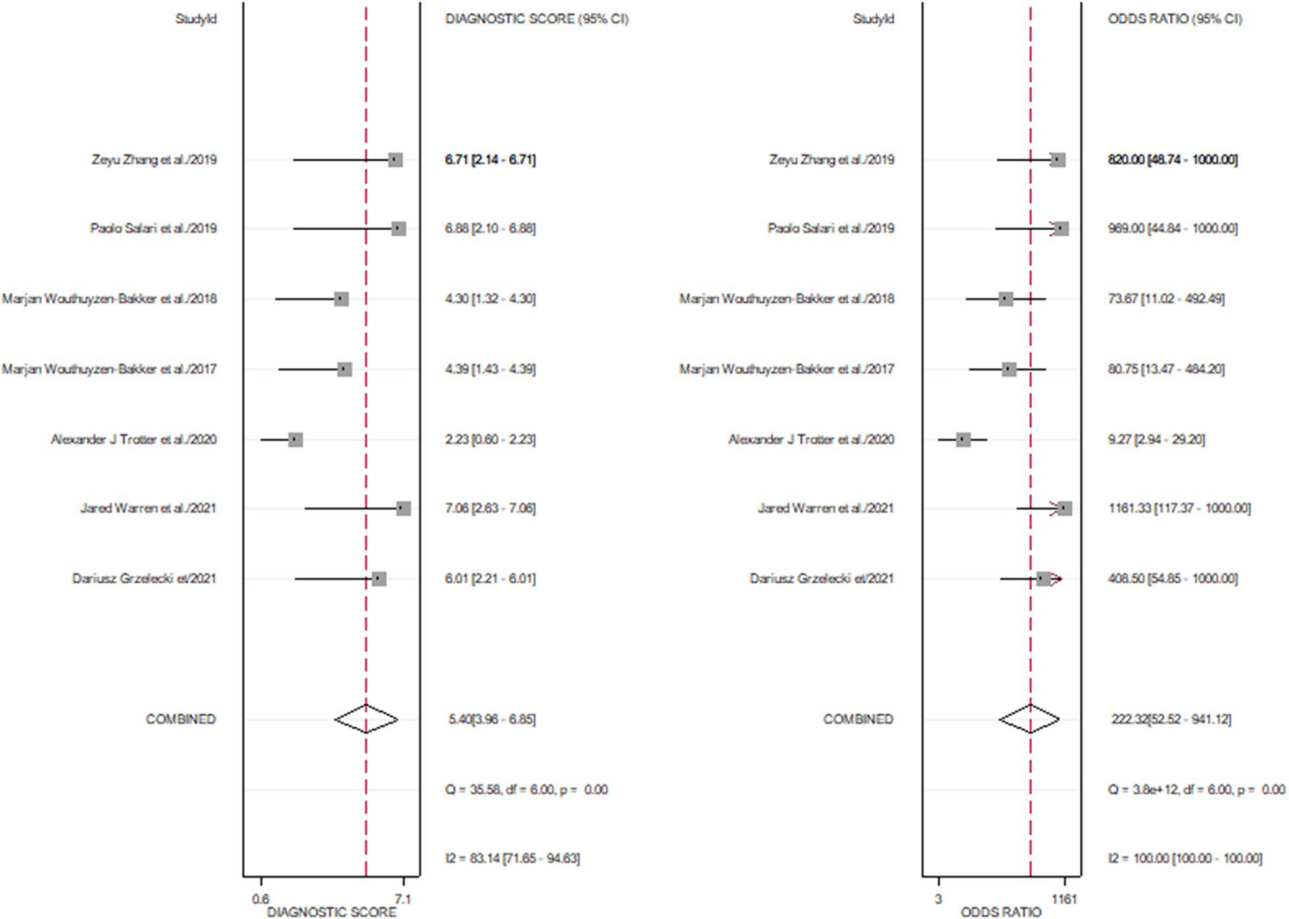
Pooled diagnostic score and diagnostic odds ratio

**Fig. 6:**
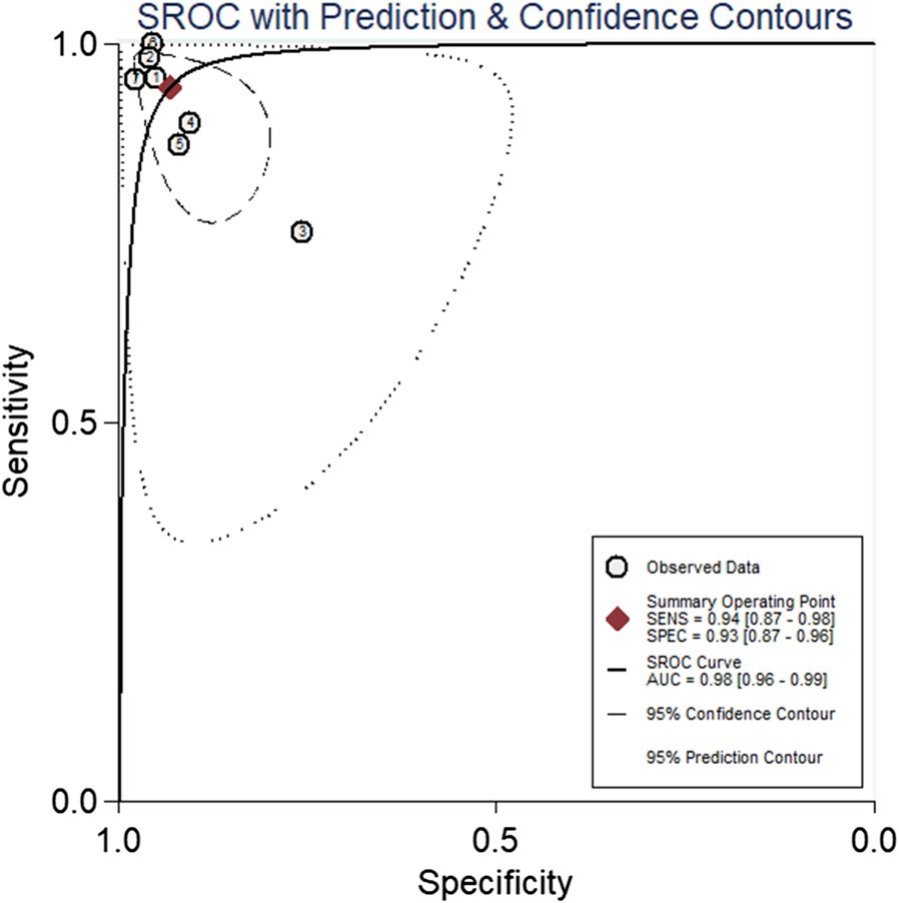
SROC curve of included studies

The pooled sensitivity of 7 studies was 0.94 (95% CI, 0.87–0.98), and the pooled specificity was 0.93 (95% CI, 0.87–0.96). The corresponding *I*^2^ statistics for sensitivity and specificity were 71.11 (95% CI, 48.63–93.58) and 76.90 (95% CI, 59.85–93.95), indicating that there was substantial heterogeneity (Fig. 4). The pooled diagnostic score and DOR were 5.4(95% CI, 3.96–6.85) and 222.32(95% CI, 52.52–941.12), respectively (Fig. 5). The area under the SROC (AUC) was 0.98 (95% CI, 0.96–0.99) (Fig. 6).

### Evaluation of clinical utility

**Fig. 7:**
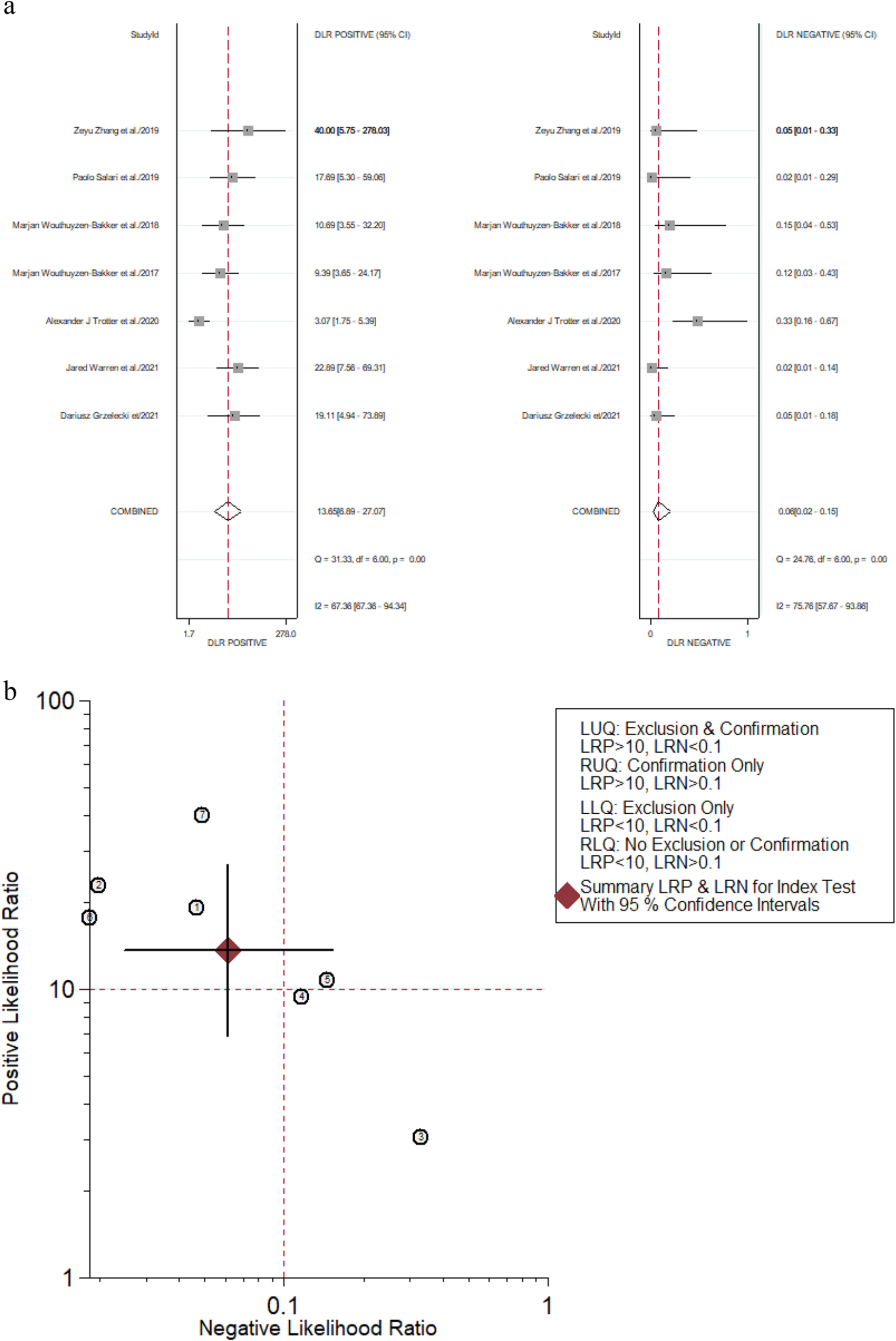
Forest plots of likelihood ratio (**a**) and likelihood ratio scatter diagrams (**b**)

**Fig. 8:**
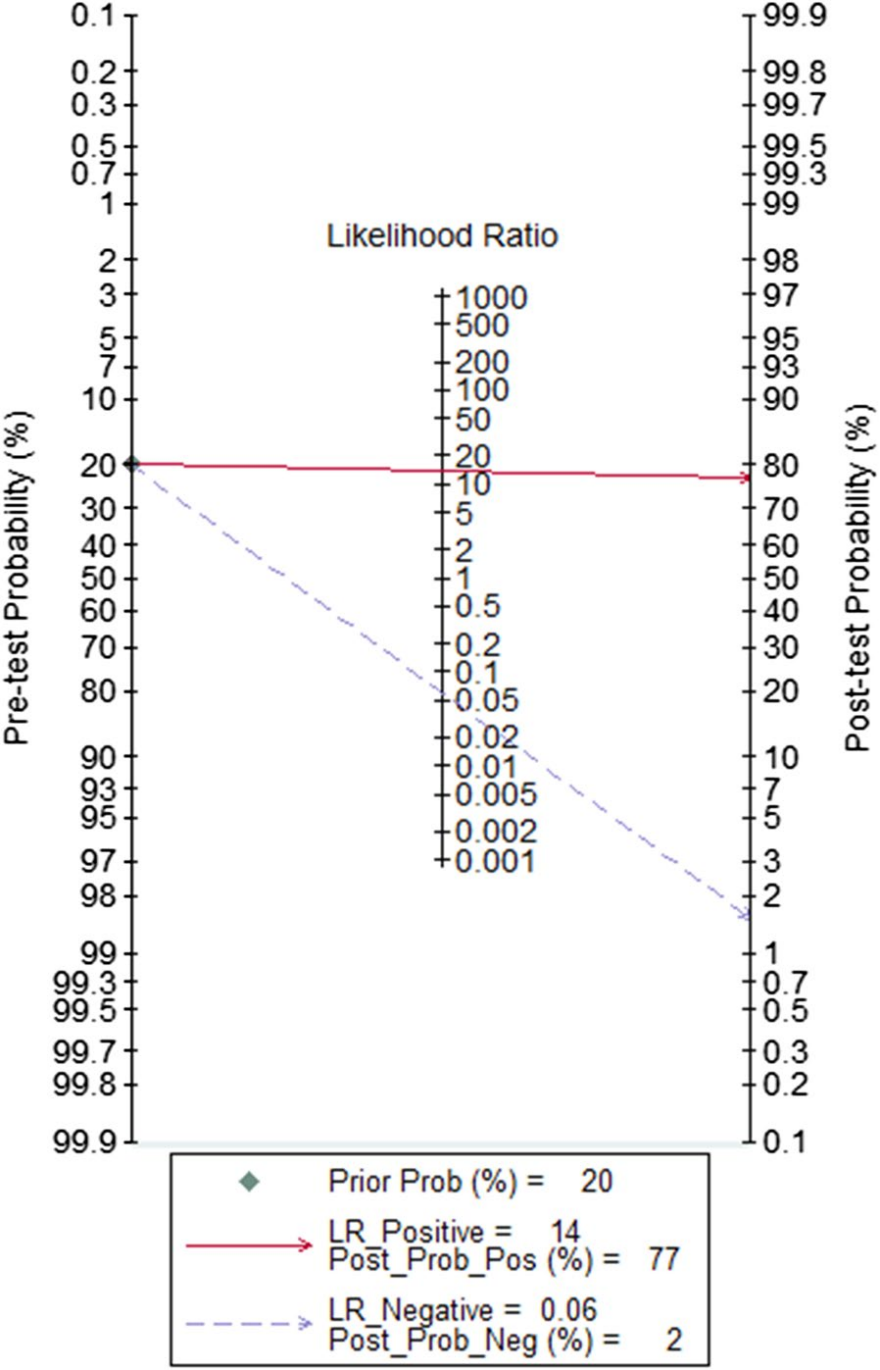
Fagan plot of the calprotectin for diagnosis of PJI

We calculated the positive likelihood ratio (PLR) and negative likelihood ratio (NLR) of calprotectin for PJI diagnosis by the forest diagram too. The pooled PLR was 13.65 (95% CI, 6.89–27.07), and the pooled NLR was 0.06 (95% CI, 0.02–0.15) (Fig. 7). When a pre-test probability was 0.2 (i.e., a 20% incidence of PJI in patients undergoing revision arthroplasty), a positive result of calprotectin for PJI diagnosis increased the probability of PJI from 20 to 77% and a negative result of calprotectin for PJI diagnosis decreased the probability of PJI to 2% presented in the Fagan plot (Fig. 8).

### Subgroup analysis

As the *I*^2^ statistics was > 50%, the result shows that there was heterogeneity between studies. We performed subgroup analysis to ascertain the potential source of heterogeneity necessarily. According to characteristics of the 7 studies, such as the study design, cutoff values, the country of study, and gold standard, we divided these studies into four subgroups based on same characteristics. In this MSIS subgroup, the overall pooled sensitivity and specificity were 0.94 (95% CI 0.86–0.98) and 0.94 (95% CI 0.89–0.97), respectively. The corresponding *I*^2^ statistics for sensitivity and specificity were 23.46 and 0.00. The corresponding *P*-value for sensitivity and specificity was 0.27 and 0.45. Results of the subgroup analysis are presented in Table 3.

**Table.3:** Subgroup analysis of calprotectin for PJI diagnosis

Subgroup analyses	No. of studies	Sensitivity (95% CI)	Specificity (95% CI)	PLR (95% CI)	NLR (95% CI)	AUC (95% CI)	Diagnostic score (95% CI)	DOR (95% CI)	*I*^2^(Sen) (95% CI)	*P*-value (Sen)	*I*^2^(Spe) (95% CI)	*P*-value (Spe)
Overall studies	7	0.94 (0.87–0.98)	0.93 (0.87–0.96)	13.7 (6.9–27.1)	0.06 (0.02–0.15)	0.98 (0.96–0.99)	5.4 (3.96–6.85)	222 (53–941)	71.11	0.00	76.90	0.00
Prospective	6	0.96 (0.91–0.98)	0.95 (0.91–0.97)	17.6 (10.5–29.4)	0.05 (0.02–0.10)	0.98 (0.96–0.99)	5.94 (4.95–6.93)	378 (141–1018)	31.00	0.20	87.49	0.00
50 mg/L	4	0.96 (0.87–0.99)	0.94 (0.89–0.97)	15.4 (8.3–28.5)	0.04 (0.01–0.15)	0.97 (0.95–0.98)	5.85 (4.29–7.40)	346 (73–1638)	54.15	0.09	0.00	0.64
Western countries	6	0.94 (0.85–0.98)	0.92 (0.85–0.96)	11.9 (5.9–24.01)	0.07 (0.02–0.18)	0.97 (0.96–0.98)	5.21 (3.65–6.77)	183 (39–871)	73.90	0.00	75.90	0.00
MSIS	4	0.94 (0.86–0.98)	0.94 (0.89–0.97)	16.3 (8.7–30.8)	0.06 (0.02–0.15)	0.98 (0.96–0.99)	5.60 (4.34–6.86)	271 (77–958)	23.46	0.27	0.00	0.45

AUC area under the curve of summary receiver-operating characteristic curves; CI confidence interval; PLR positive likelihood ratio; NLR negative likelihood ratio; DOR diagnostic odds ratio; Sen = sensitivity; Spe = specificity

## Discussion

Total joint arthroplasty (TJA) is the symbol of great progress for modern medical, because it can greatly relieve the pain of the patient, make restricted function to recover and improve the quality of life [27]. But PJI, a terrified complication after TJA, can be challenging and difficult to accurately diagnose and manage with effect for most surgeons [28, 29]. It is very vital for the management of PJI to distinguish between PJI and aseptic loosening in the face of the painful joint after arthroplasty [30, 31]. Orthopedic surgeons diagnose PJI according to the clinical manifestations, biomarkers from blood and synovial fluid, the microbiological results, molecular diagnosis, histopathology, and imaging results [32]. Biomarkers from blood and synovial fluid provide diagnose PJI with material evidence. For a host of researchers, much attention has been paid to discovering a novel and promising biomarker that has the potential to be a biomarker for diagnostic criteria compared with current ones [33]. Many researches show that synovial biomarkers are more useful than those from blood at accuracy diagnosis of PJI, such as α-defensin, interleukin-6, interleukin-1β, leukocyte esterase, and interleukin-17 [11, 34].

Calprotectin from synovial fluid has been regarded as a promising test for diagnosis according to many studies [25, 35]. M. Wouthuyzen-Bakker et al. conducted a first study on diagnostic value of synovial calprotectin that a cutoff value of 50 mg/L has high sensitivity (89%) and specificity (90%), with a NPV of 97%. This study shows that calprotectin may become a promising test to exclude PJI [24]. D. Grzelecki et al. deemed that calprotectin was superior to CRP, ESR, interleukin-6 (IL-6), and leucocyte esterase (LE) on diagnostic value of chronic PJI [22]. However, there is not a meta-analysis on calprotectin in the diagnosis of PJI hitherto, and therefore it is very necessary for many researchers to analyze its diagnostic value.

The meta-analysis including 7 studies shows that the calprotectin has excellent PJI diagnostic value, with high sensitivity (94%) and specificity (93%). But there was a great heterogeneity in this study, because the corresponding *I*^2^ statistics for sensitivity and specificity was 71.11 and 76.9, respectively. The AUC for pooled data from the 7 studies was 0.98, demonstrating excellent diagnostic accuracy to identify PJI due to the AUC > 0.97 [36]. The DOR of this meta-analysis was 222.32 (95% CI, 52.52–941.12) due to > 10, showing that calprotectin may serve as a good predictor for PJI. The PLR and the NLR of calprotectin were 13.65 (95% CI, 6.89–27.07) and 0.06 (95% CI, 0.02–0.15), respectively. It is indicated that calprotectin could be a biomarker of moderate diagnostic value in PJI, as the PLR was > 10 and the NLR was < 0.1. Besides, the Fagan plot diagram revealed that calprotectin was a promising and valuable biomarker for predicting and excluding PJI.

The C-reactive has been included in the diagnostic criteria in the 2018 ICM. Chi Wang et al. reported the pooled sensitivity and specificity for C-reactive were 0.92 (95% CI 0.86–0.96) and 0.90 (95% CI 0.87–0.93), respectively [37]. The pooled sensitivity and specificity of our meta-analysis are greater than the results done by Chi Wang et al. The American Academy of Orthopaedic Surgeons Clinical Practice Guideline “Diagnosis and Prevention of Periprosthetic Joint Infections” showed that α-defensin testing was good for the diagnosis of PJI [38]. The results of the study done by Giovanni Balato et al. showed that the pooled sensitivity and specificity for α-defensin were 0.90 (95% CI 0.83–0.94) and 0.95 (95% CI 0.92–0.96), respectively [39]. The pooled sensitivity of our meta-analysis is greater than the results done by Giovanni Balato et al., but the pooled specificity is less than the results done by Giovanni Balato et al. So calprotectin was a valuable biomarker for diagnosis of PJI compared with α-defensin and C-reactive.

Given remarkable heterogeneity from sensitivity and specificity, we must investigate the sources of heterogeneity in this study by subgroup analysis. One of the main sources of heterogeneity is the threshold effect for the diagnostic meta-analysis. Therefore, we divided four studies that regarded 50 mg/L as the diagnostic threshold of PJI as a subgroup. In this subgroup analysis, the AUC of 50 mg/L subgroup was 0.97 and less than the AUC (0.98) of overall studies. The corresponding *I*^2^ statistics for sensitivity was 54.15 (> 50), indicating that there was still heterogeneity. Next, we explored other potential causes of heterogeneity such as diagnostic standard, study design, and the country of study by subgroup analysis. Fortunately, we found finally that the sources of heterogeneity of the meta-analysis about calprotectin were originated from the diagnostic standard. The AUC and NLR of the MSIS subgroup were completely consistent with those of all studies. In addition, the corresponding *I*^2^ statistics for sensitivity and specificity was 23.46 and 0.00, (*I*^2^ < 50), respectively. The pooled sensitivity, specificity, PLR, diagnostic score, and DOR of the MSIS subgroup were greater than or equal to the overall studies.

The costs of synovial calprotectin test for PJI are relatively cheap compared with interleukin-6 and alpha-defensin measured with lateral flow immunoassays [40, 41]. It is available for majority hospitals to use calprotectin as biomarker of diagnosing PJI [42].

There are several limitations in the meta-analysis. First, this study includes the limited number of articles, and the included sample size is limited. So, it is very difficult for us to discover other potential heterogeneity by conducting further subgroup analyses. Second, there is no internationally recognized gold standard for diagnosis of PJI, and this meta-analysis was used in multiple reference standards. Third, the studies included in this meta-analysis used tests dedicated to different samples (fecal, synovial fluid, plasma). This issue may influence on the received results and cutoff values. Finally, this meta-analysis lacks more studies about different calprotectin cutoff values. However, our subgroup analysis shows that there is no remarkable threshold effect.

## Conclusion

We first evaluate diagnostic value about synovial calprotectin by meta-analysis. Our results reveal that synovial calprotectin for PJI has good diagnostic accuracy due to the high sensitivity and specificity. Besides, synovial calprotectin can serve as a significant tool to exclude PJI based on its low NLR (0.06) of overall pooled data. In spite of requiring more researches about calprotectin, we still deem that synovial calprotectin will serve as a promising biomarker for diagnosis of PJI because of its advantages, such as high diagnostic accurate and convenience.

## Supplementary Information

**Additional file 1**. Search strategy.

## Data Availability

The datasets used and/or analyzed during the current study are not publicly available due to feasibility but are available from the corresponding author on reasonable request.

## Abbreviations

PJI: Periprosthetic joint infection
AAOS: The American Academy of Orthopedic Surgeon
MSIS: Musculoskeletal Infection Society
ICM: International Consensus Meeting
IDSA: Infectious Diseases Society of America
LR: Likelihood ratio
DOR: Diagnostic odds ratio
SROC: Summary receiver operating characteristic
AUC: Area under the curve
PRISMA: Preferred Reporting Items for Systematic Reviews and Meta-Analyses

## References

[1] Kurtz SM, Ong KL, Schmier J, Zhao K, Mowat F, Lau E (2009). Primary and revision arthroplasty surgery caseloads in the United States from 1990 to 2004. J Arthroplasty.

[2] Hartzler MA, Li K, Geary MB, Odum SM, Springer BD (2020). Complications in the treatment of prosthetic joint infection. Bone Joint J.

[3] Wyatt MC, Beswick AD, Kunutsor SK, Wilson MJ, Whitehouse MR, Blom AW (2016). The Alpha-Defensin immunoassay and leukocyte esterase colorimetric strip test for the diagnosis of periprosthetic infection: a systematic review and Meta-Analysis. J Bone Joint Surg.

[4] Kurtz SM, Lau E, Watson H, Schmier JK, Parvizi J (2012). Economic burden of periprosthetic joint infection in the United States. J Arthroplasty.

[5] Bozic KJ, Ries MD (2005). The impact of infection after total hip arthroplasty on hospital and surgeon resource utilization. J Bone Joint Surg Am.

[6] Haddad FS, Ngu A, Negus JJ (2017). Prosthetic joint infections and cost analysis?. Adv Exp Med Biol.

[7] Parvizi J, Ghanem E, Menashe S, Barrack RL, Bauer TW (2006). Periprosthetic infection: What are the diagnostic challenges?. J Bone Joint Surg Am.

[8] Parvizi J, Della VC (2010). AAOS Clinical Practice Guideline: diagnosis and treatment of periprosthetic joint infections of the hip and knee. J Am Acad Orthop Surg.

[9] Tande AJ, Patel R (2014). Prosthetic joint infection. Clin Microbiol Rev.

[10] Chisari E, Parvizi J (2020). Accuracy of blood-tests and synovial fluid-tests in the diagnosis of periprosthetic joint infections. Expert Rev Anti Infect Ther.

[11] Deirmengian C, Kardos K, Kilmartin P, Cameron A, Schiller K, Parvizi J (2014). Diagnosing periprosthetic joint infection: has the era of the biomarker arrived?. Clin Orthop Relat Res.

[12] Lee YS, Koo KH, Kim HJ, Tian S, Kim TY, Maltenfort MG, Chen AF (2017). Synovial fluid biomarkers for the diagnosis of periprosthetic joint infection: a systematic review and meta-analysis. J Bone Joint Surg Am.

[13] Edgeworth J, Gorman M, Bennett R, Freemont P, Hogg N (1991). Identification of p8,14 as a highly abundant heterodimeric calcium binding protein complex of myeloid cells. J Biol Chem.

[14] McInnes IB, Schett G (2011). The pathogenesis of rheumatoid arthritis. N Engl J Med.

[15] Perera C, McNeil HP, Geczy CL (2010). S100 Calgranulins in inflammatory arthritis. Immunol Cell Biol.

[16] Wang Y, Liang Y (2019). Clinical significance of serum calprotectin level for the disease activity in active rheumatoid arthritis with normal C-reactive protein. Int J Clin Exp Pathol.

[17] Jukic A, Bakiri L, Wagner EF, Tilg H, Adolph TE (2021). Calprotectin: From biomarker to biological function. Gut.

[18] Ma Y, Fan D, Xu S, Deng J, Gao X, Guan S, Pan F (2020). Calprotectin in spondyloarthritis: a systematic review and meta-analysis. Int Immunopharmacol.

[19] Trotter AJ, Dean R, Whitehouse CE, Mikalsen J, Hill C, Brunton-Sim R, Kay GL, Shakokani M, Durst A, Wain J, McNamara I, O'Grady J (2020). Preliminary evaluation of a rapid lateral flow calprotectin test for the diagnosis of prosthetic joint infection. Bone Joint Res.

[20] Liberati A, Altman DG, Tetzlaff J, Mulrow C, Gøtzsche PC, Ioannidis JP, Clarke M, Devereaux PJ, Kleijnen J, Moher D (2009). The PRISMA statement for reporting systematic reviews and meta-analyses of studies that evaluate healthcare interventions: explanation and elaboration. BMJ.

[21] Zhang Z, Cai Y, Bai G, Zhang C, Li W, Yang B, Zhang W (2020). The value of calprotectin in synovial fluid for the diagnosis of chronic prosthetic joint infection. Bone Joint Res.

[22] Grzelecki D, Walczak P, Szostek M, Grajek A, Rak S, Kowalczewski J (2021). Blood and synovial fluid calprotectin as biomarkers to diagnose chronic hip and knee periprosthetic joint infections. Bone Joint J.

[23] Warren J, Anis HK, Bowers K, Pannu T, Villa J, Klika AK, Colon-Franco J, Piuzzi NS, Higuera CA (2021). Diagnostic utility of a novel point-of-care test of calprotectin for periprosthetic joint infection after total knee arthroplasty: a prospective cohort study. J Bone Joint Surg Am.

[24] Wouthuyzen-Bakker M, Ploegmakers J, Kampinga GA, Wagenmakers-Huizenga L, Jutte PC, Muller KA (2017). Synovial calprotectin: a potential biomarker to exclude a prosthetic joint infection. Bone Joint J.

[25] Wouthuyzen-Bakker M, Ploegmakers J, Ottink K, Kampinga GA, Wagenmakers-Huizenga L, Jutte PC, Kobold A (2018). Synovial calprotectin: an inexpensive biomarker to exclude a chronic prosthetic joint infection. J Arthroplasty.

[26] Salari P, Grassi M, Cinti B, Onori N, Gigante A (2020). Synovial fluid calprotectin for the preoperative diagnosis of chronic periprosthetic joint infection. J Arthroplasty.

[27] Bumpass DB, Nunley RM (2012). Assessing the value of a total joint replacement. Curr Rev Musculoskelet Med.

[28] Vielgut I, Sadoghi P, Wolf M, Holzer L, Leithner A, Schwantzer G, Poolman R, Frankl B, Glehr M (2015). Two-stage revision of prosthetic hip joint infections using antibiotic-loaded cement spacers: when is the best time to perform the second stage?. Int Orthop.

[29] Osmon DR, Berbari EF, Berendt AR, Lew D, Zimmerli W, Steckelberg JM, Rao N, Hanssen A, Wilson WR (2013). Executive summary: diagnosis and management of prosthetic joint infection: clinical practice guidelines by the Infectious Diseases Society of America. Clin Infect Dis.

[30] Garvin KL, Konigsberg BS (2011). Infection following total knee arthroplasty: prevention and management. J Bone Joint Surg Am.

[31] Parvizi J, Adeli B, Zmistowski B, Restrepo C, Greenwald AS (2012). Management of periprosthetic joint infection: the current knowledge: AAOS exhibit selection. J Bone Joint Surg Am.

[32] Cataldo MA, Petrosillo N, Cipriani M, Cauda R, Tacconelli E (2010). Prosthetic joint infection: recent developments in diagnosis and management. J Infect.

[33] Keemu H, Vaura F, Maksimow A, Maksimow M, Jokela A, Hollmén M, Mäkelä K (2021). Novel biomarkers for diagnosing periprosthetic joint infection from synovial fluid and serum. JB JS Open Access.

[34] Saleh A, Ramanathan D, Siqueira M, Klika AK, Barsoum WK, Rueda C (2017). The diagnostic utility of synovial fluid markers in periprosthetic joint infection: a systematic review and meta-analysis. J Am Acad Orthop Surg.

[35] Wasterlain AS, Goswami K, Ghasemi SA, Parvizi J (2020). Diagnosis of periprosthetic infection: recent developments. J Bone Joint Surg Am.

[36] Jones CM, Athanasiou T (2005). Summary receiver operating characteristic curve analysis techniques in the evaluation of diagnostic tests. Ann Thorac Surg.

[37] Wang C, Wang Q, Li R, Duan JY, Wang CB (2016). Synovial fluid c-reactive protein as a diagnostic marker for periprosthetic joint infection: a systematic review and meta-analysis. Chin Med J (Engl).

[38] Chen AF, Riedel S (2020). A case illustrating the practical application of the AAOS clinical practice guideline: diagnosis and prevention of periprosthetic joint infection. J Am Acad Orthop Surg.

[39] Balato G, de Matteo V, Ascione T, Di Donato SL, De Franco C, Smeraglia F, Baldini A, Mariconda M (2020). Laboratory-based versus qualitative assessment of α-defensin in periprosthetic hip and knee infections: a systematic review and meta-analysis. Arch Orthop Trauma Surg.

[40] Bauer TW (2017). CORR insights(®): How reliable is the Alpha-Defensin immunoassay test for diagnosing periprosthetic joint infection? A prospective study. Clin Orthop Relat Res.

[41] Wimmer MD, Ploeger MM, Friedrich MJ, Bornemann R, Roessler PP, Gravius S, Randau TM (2016). The QuickLine IL-6 lateral flow immunoassay improves the rapid intraoperative diagnosis of suspected periprosthetic joint infections. Technol Health Care.

[42] Jonsson N, Nilsen T, Gille-Johnson P, Bell M, Martling CR, Larsson A, Mårtensson J (2017). Calprotectin as an early biomarker of bacterial infections in critically ill patients: an exploratory cohort assessment. Crit Care Resusc.

